# The effect of hot days on occupational heat stress in the manufacturing industry: implications for workers’ well-being and productivity

**DOI:** 10.1007/s00484-018-1530-6

**Published:** 2018-03-30

**Authors:** Tjaša Pogačar, Ana Casanueva, Katja Kozjek, Urša Ciuha, Igor B. Mekjavić, Lučka Kajfež Bogataj, Zalika Črepinšek

**Affiliations:** 1 Biotechnical Faculty, University of Ljubljana, Jamnikarjeva 101, 1000 Ljubljana, Slovenia; 2 Federal Office of Meteorology and Climatology, MeteoSwiss, Zurich Airport, Zurich, Switzerland; 3 Slovenian Environmental Agency, Vojkova 1b, Ljubljana, Slovenia; 4 Department of Automation, Biocybernetics and Robotics, Jozef Stefan Institute, Jamova cesta 39, 1000 Ljubljana, Slovenia; 5 Department of Biomedical Physiology and Kinesiology, Simon Fraser University, Burnaby, British Columbia V5A 1S6 Canada

**Keywords:** Heat stress, Hot day, Wet Bulb Globe Temperature, Occupational health, Climate change

## Abstract

Climate change is expected to exacerbate heat stress at the workplace in temperate regions, such as Slovenia. It is therefore of paramount importance to study present and future summer heat conditions and analyze the impact of heat on workers. A set of climate indices based on summer mean (Tmean) and maximum (Tmax) air temperatures, such as the number of hot days (HD: Tmax above 30 °C), and Wet Bulb Globe Temperature (WBGT) were used to account for heat conditions in Slovenia at six locations in the period 1981–2010. Observed trends (1961–2011) of Tmean and Tmax in July were positive, being larger in the eastern part of the country. Climate change projections showed an increase up to 4.5 °C for mean temperature and 35 days for HD by the end of the twenty-first century under the high emission scenario. The increase in WBGT was smaller, although sufficiently high to increase the frequency of days with a high risk of heat stress up to an average of a third of the summer days. A case study performed at a Slovenian automobile parts manufacturing plant revealed non-optimal working conditions during summer 2016 (WBGT mainly between 20 and 25 °C). A survey conducted on 400 workers revealed that 96% perceived the temperature conditions as unsuitable, and 56% experienced headaches and fatigue. Given these conditions and climate change projections, the escalating problem of heat is worrisome. The European Commission initiated a program of research within the Horizon 2020 program to develop a heat warning system for European workers and employers, which will incorporate case-specific solutions to mitigate heat stress.

**Electronic supplementary material:**

The online version of this article (10.1007/s00484-018-1530-6) contains supplementary material, which is available to authorized users.

## Introduction

Humans have inhabited all regions of the globe, as a result of their ability to adapt to a wide range of environmental conditions and also due to their ingenuity in developing shelters and clothing to protect them from environmental stressors. This ability to adapt to extreme temperature events is vital for the survival of humans (Basarin et al. 2016) and will be of particular importance during this century. Namely, the nonlinearity expected with further warming will cause the probability of hot extremes (e.g., heat waves) at the projected 2 °C warming to double that for the projections of 1.5 °C global warming (Fischer and Knutti 2015). Global warming must also be considered an occupational health hazard for individuals working in both outdoor or indoor conditions without effective climate protection and/or control (Parsons 2014).

It is now beyond doubt that global warming has increased the number of heat waves experienced in Europe. These are becoming longer in duration and greater in magnitude (IPCC 2013). The effect of climate change in general, and heat waves in particular, on weather patterns, crops, and livestock have already been demonstrated (e.g., Fischer et al. 2007; Aggarwal and Upadhyay 2013; Pagani et al. 2017). The risk of heat stress should not be underestimated in temperate regions, especially when dealing with unacclimatized workers being exposed to heat waves (Adam-Poupart et al. 2013). Short-term heat acclimatization usually takes 3–12 days, but complete (long-term) acclimatization to an unfamiliar thermal environment may take several years (Koppe et al. 2004). The problems of high ambient temperatures are augmented in urban areas, both due to the absorption of thermal energy by the buildings and roads, which also has a greater impact on the population (Pascal et al. 2013), and this can be enhanced in various working environments. Occupational exposure to heat without sufficient protection may not only increase the risk of heat-related illnesses and injuries (Almeida et al. 2013; Smith et al. 2014) but also compromise economic productivity by reducing work efficiency (Franchetti and Komaki 2012; Kosonen and Tan 2004). High body temperature, particularly when combined with dehydration, causes heat exhaustion, heat stroke, and, in extreme cases, death. A worker’s natural response to prevent the risk of hyperthermia is to reduce work intensity and/or limit working hours, thus minimizing heat production in the former and reducing heat exposure in the latter case. Both of these strategies reduce productivity and economic output (Parsons 2014).

Heat-related illnesses and injuries are largely avoidable, with appropriate heat warning systems or mitigation strategies (Pascal et al. 2013). Key components of any heat prevention plan are the appropriate choice of indices of heat stress, thresholds of these indices that define critical events, and, most importantly, accurate forecasting of such critical events. Generally, excessive heat creates occupational health risks and reduces work capacity and labor productivity at temperatures above 35 °C (Parsons 2014), but ambient relative humidity also has to be taken into account. These two parameters can be combined in heat stress indices, such as the Wet Bulb Globe Temperature (WBGT, Budd 2008).

Heat stress, as a consequence of global warming, is projected to monotonically increase throughout the twenty-first century in many regions of the world (Fischer and Schär 2010; Fischer et al. 2012; Pal and Eltahir 2015; Suzuki-Parker and Kusaka 2016). Under certain conditions, such as manual labor during heat waves, the capacity of the human body to dissipate adequate heat may be anticipated on a regular basis in certain parts of the world during this century (Smith et al. 2014).

The European Commission has recently initiated a program of research (the HEAT-SHIELD project) to assess the effect of the present and projected changes in climate on workers in five strategic industries (transport, construction, manufacturing, tourism, and agriculture) representing approximately 40% of the workforce in Europe. The aim of the initiative is to assess the effect of heat waves on the health and well-being of workers and on their ability to work. The demonstrated loss in productivity will hopefully provide the impetus to develop strategies to increase the resilience of workers during the anticipated changes in climate. This study analyses the present and projected local climate conditions in Slovenia and, in particular, at an automobile parts manufacturing plant in Slovenia and assesses how the summer heat waves of 2016 were experienced by the workforce.

## Methods

The purpose of the study was to assess present climate conditions and examine future climate change projections in Slovenia, focusing on the local climate conditions in the region of the odelo d.o.o. manufacturing plant (Prebold, Slovenia). The analysis of the local climate conditions focused on the pattern of the summer heat waves of 2016. The study also surveyed the workers in the odelo d.o.o. manufacturing plant regarding on how the heat waves affected them.

### Present and future summer heat conditions in Slovenia

**Fig. 1:**
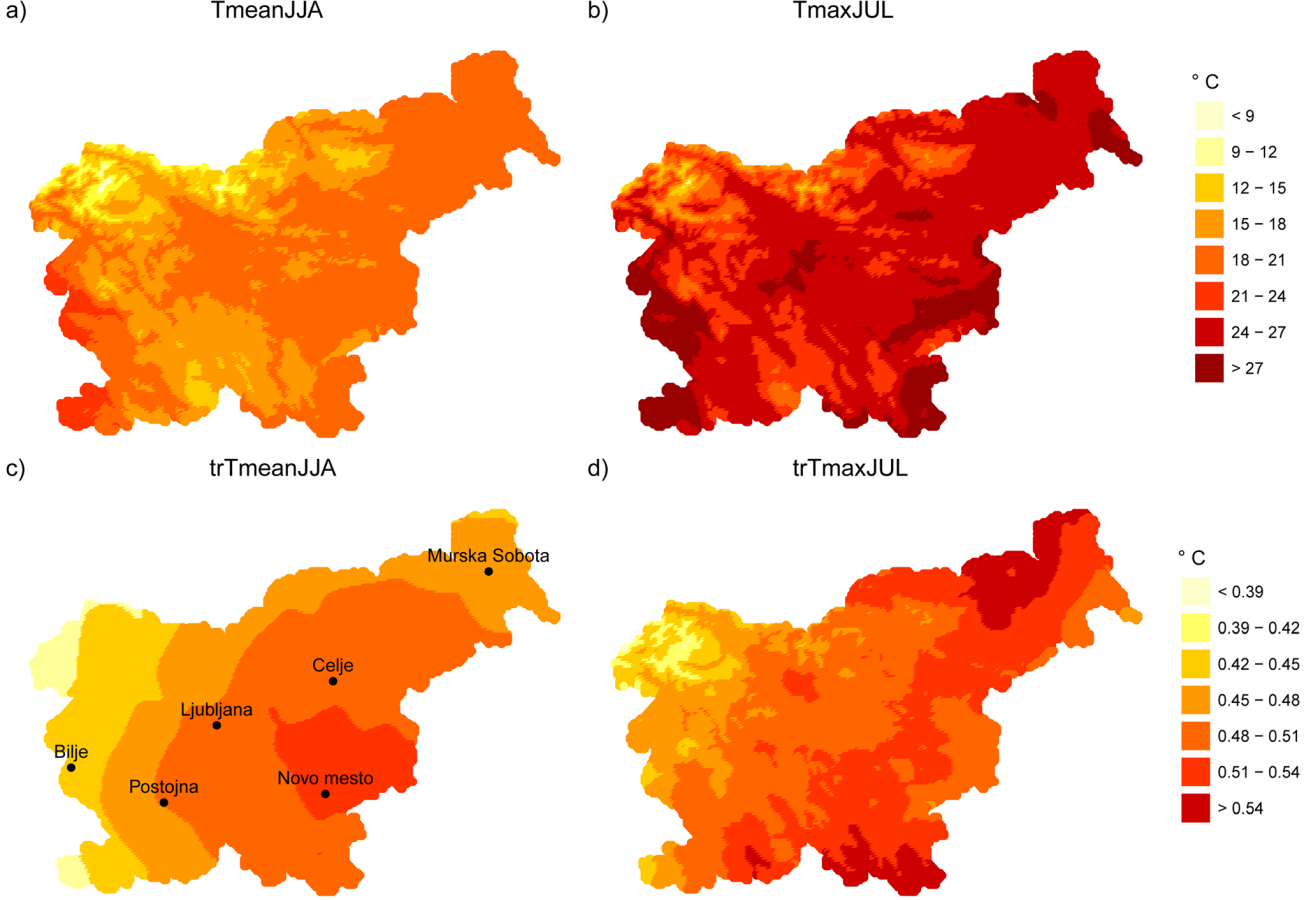
Maps showing the spatial distribution of 30-year (1981–2010) mean summer temperature (**a**), 30-year (1981–2010) mean maximum temperature of the hottest month (July) (**b**), 51-year (1961–2011) long-term trend (per decade) of mean summer temperature (**c**), and 51-year (1961–2011) long-term trend (per decade) of mean maximum temperature of the hottest month (**d**)

Mean and extreme heat conditions were analyzed from the daily mean (Tmean) and maximum (Tmax) temperatures available from the Slovenian Environmental Agency. Data from 60 weather stations were used to build a gridded map based on the homogenized time series of daily means and maxima to account for past heat conditions. The horizontal resolution of the grid was 1 × 1 km, which accounted for 20,916 points over the entire region of Slovenia. A set of 16 point stations well-distributed across Slovenia were considered for the climate change projections, with special focus on six locations (Fig. 1c): Bilje (55 m above sea level (a.s.l.), Ljubljana (299 m a.s.l.), Celje (244 m a.s.l.), Murska Sobota (188 m a.s.l.), Novo mesto (220 m a.s.l.), and Postojna (533 m a.s.l.).

Two different periods were considered for the calculation of the climate indices: 1961–2011 and 1981–2010. For the heat stress index calculation, daily mean dew point temperature values were also considered for the point stations.

#### Climate indices

Summer temperature conditions in Slovenia can be described with distribution maps of different summer temperature indices. Mean summer (June–August: TmeanJJA) temperature, monthly mean of the daily maximum temperature of the hottest month (July: TmaxJUL), and long-term trends of both previous variables were the key variables chosen for this purpose (Kozjek et al. 2017a, b). Additionally, we also used the number of hot days (HD), defined as the number of summer days with daily maximum temperatures above 30 °C, in accordance with climatological practice (e.g., Kysely 2010). Data from the 30-year period (1981–2010) were used for calculating temperature means, and the 51-year period (1961–2011) was used for calculating long-term trends. This non-standard 51-year time period was used since it was assigned in the project “Climate variability of Slovenia” (Vertačnik et al. 2015). The 30-year (1981–2010) averages of TmeanJJA, TmaxJUL, and HD were considered as the final indices, whereas their trends trTmeanJJA, trTmaxJUL, and trHD were obtained from the 51-year period 1961–2011 using the Theil-Sen method (Theil 1950; Sen 1968). In a subsequent analysis, the relationship between the number of hot days and average summer temperatures was fitted with the linear and quadratic trend.

In addition to temperature-based indices, we also used Wet Bulb Globe Temperature (WBGT) as the heat stress index. WBGT combines the effect of temperature and humidity and is recommended by the International Standard Organization (ISO) as occupational heat stress index. There are more sophisticated indices and models which consider the impact of clothing and activity, but all indices have advantages and disadvantages. Within the framework of the Horizon 2020 HEAT-SHIELD project (https://www.heat-shield.eu/), the WBGT was selected (for example, used also in Kjellstrom et al. (2017)), because (1) it is the most widely used index to assess heat stress on working people which was the final aim of the work, (2) it can be calculated from standard meteorological parameters, and (3) it can be easily interpreted by means of international standards (ISO 1989, 2017). Daily values of WBGT were obtained according to Bernard and Pourmoghani (1999), assuming no strong source of radiation (inside the factory), and setting wind speed to 1 m/s (equivalent to slow walk or movement of arms and legs during work), as recommended by Lemke and Kjellstrom (2012). WBGT was calculated from daily maximum temperature and daily mean dew point, to target the highest heat stress of the day. In addition to summer mean values of WBGT (WBGTmean), the summer maximum (WBGTx) and the number of summer days with WBGT above 27 °C (WBGTg27) were analyzed to account for extreme heat stress conditions. The 27 °C WBGT threshold is associated with high heat stress risk for acclimatized workers doing moderate work (NIOSH 2016). As for the temperature-based indices, the multi-year averages are presented as final values.

All selected indices and their description are presented in Table 1.

**Table 1:** Description of selected temperature-derived indices

Variable	Symbol	Description	Unit
Tmean	TmeanJJA	Summer mean temperature	°C
	trTmeanJJA	Summer trend of TmeanJJA	°C/decade
Tmax	TmaxJUL	Monthly (July) mean of daily maximum temperature	°C
	HD	Summer hot days (i.e., number of summer days with Tmax ≥ 30)	days
	trTmaxJUL	Summer trend of TmaxJUL	°C/decade
	trHD	Summer trend of HD	days/decade
WBGT	WBGTmean	Summer mean WBGT	°C
	WBGTx	Summer maximum WBGT	°C
	WBGTg27	Number of summer days with WBGT > 27 °C	days

#### Spatial interpolation

As mentioned above, gridded observational data were used to illustrate past heat conditions in Slovenia (TmeanJJA, TmaxJUL, trTmeanJJA, trTmaxJUL). Spatial interpolation of climate variables and their long-term trends into a regular grid was performed using residual kriging (Cressie 1993). The value in each grid point was calculated using the data from surrounding meteorological stations and selected geographical variables, which describe the spatial variability of an interpolated variable with statistical significance. In addition to data from the Slovene measuring network, the data from near-border meteorological stations in Italy, Austria, and Croatia were also used. The detailed methodology is described in Tveito (2008) and Dolinar (2016). Interpolated results were validated using “leave one out” cross-validation methodology (Cressie 1993). The normalized root mean square prediction error (RMSEr, see Eq. 1), a measure of the normalized accuracy of spatial interpolation prediction, varied from 13 to 14% for monthly air temperature grids. RMSEr can be considered satisfactory, if it is less than 40% (Hengl 2007). For all variables, the gridded values have the highest uncertainty in regions with altitudes above 1000 m, since the input data density in this region is very poor (Dolinar 2016; Tveito 2008). With gridded values of all the variables, distribution maps of each variable were plotted using ggplot2 package from R-software, for the entire territory of Slovenia.

1 $$ \mathrm{RMSEr}=\frac{\mathrm{RMSE}}{\mathrm{SD}} $$

where RMSE is the root mean square error and SD is the standard deviation of point measurements.

#### Climate model data and bias correction

Regional climate models (RCMs) are the most widely used tools to provide climate change projections on a regional scale. They numerically solve the governing equations of the atmosphere in a limited spatial domain, subject to initial and boundary conditions taken from global climate models (GCMs). In the present work, a comprehensive set of RCM simulations from the EURO-CORDEX initiative (the European branch of CORDEX, Jacob et al. 2014; Kotlarski et al. 2014) were considered, to project future heat conditions in Slovenia. In particular, we used 84 simulations (RCM-GCM chains, see Online Resource 1) spanning different combinations of RCMs and GCMs, at two horizontal resolutions (0.11° and 0.44°, approximately 12 and 50 km) covering three representative concentration pathways (RCP2.6, RCP4.5, and RCP8.5, from low to high forcing scenarios). We considered daily mean, maximum, and dew point temperatures (the latter calculated from relative humidity and mean temperature) to obtain the temperature- and WBGT-derived indices for the period 1981–2010 (as a reference period) and for the end of the twenty-first century (2070–2099).

Despite the tremendous advances in regional climate modeling, RCMs are prone to systematic biases and their spatial resolution is still too coarse to be used in impact studies. Therefore, a statistical downscaling and bias correction method was applied to the RCMs to overcome these limitations. The selected method was the empirical quantile mapping (QM, Panofsky and Brier 1968; Déqué 2007; Rajczak et al. 2016), which consists of matching the simulated and observed distributions by establishing a quantile-dependent correction function, between the observed and simulated quantiles. The corrections are performed independently for each variable, as commonly done in climate sciences (Casanueva et al. 2018a), but the inter-variable relationships from the original simulations are preserved (Wilcke et al. 2013). For more details on the RCM data and bias correction approach, the reader is referred to Casanueva et al. 2018b.

### Case study in the odelo d.o.o. company

The case study was performed in the odelo d.o.o. company located in Prebold (Slovenia), 13 km from Celje. The company produces automotive rear lights using injection molding. Measurements of air temperature at 1.5 and 0.05 m height and relative humidity at 1.5 m height at this workplace was made at 15-min intervals using temperature and humidity sensors (Modular Sensor Recorders, MSR, Switzerland) and stored on a data cloud. Recording of internal ambient conditions in the manufacturing halls commenced in June 2016.

A survey regarding thermal comfort and temperature sensation due to heat stress was conducted among 400 workers in odelo in August 2016, adopting the approach of the Hothaps (The High Occupational Temperature, Health, and Productivity Suppression) Program (Kjellstrom et al. 2011). The workforce comprised a greater number of females and consequently, also more females participated in the survey: 27 women and 14 men above 50 years old and 182 women and 177 men under or equal to 50 years old. They were instructed to answer the questions about working conditions during heat waves.

## Results

### Past and present summer heat conditions in Slovenia

The most recent climate classification of Slovenia divides the country into six regions based on 31 meteorological variables (Kozjek et al. 2017a): sub-Mediterranean climate region (Bilje), wet climate of hilly region, moderate climate of hilly region (Postojna), subcontinental climate region (Ljubljana, Celje, Murska Sobota, Novo mesto), subalpine climate region, and Alpine climate region. Summer maximum air temperatures do not reach extremely high values in Alpine and subalpine regions and in the wet climate of the hilly region. In agreement with ARSO (2017), averaged summer air temperature (TmeanJJA) has a great variability across Slovenia, from around 8 °C to more than 20 °C with maximum values in northeastern and southwestern part of Slovenia (Fig. 1a). The largest positive trend (trTmeanJJA) is clearly in the southeastern part, reaching over 0.5 °C per decade (Fig. 1c). The geographical distribution of the 30-year average of maximum air temperatures in July (TmaxJUL) is very similar to the one of mean summer temperature (Fig. 1b). Additionally, there are very high maximum temperatures in the southeastern part. The highest trend of maximum air temperatures in July (trTmaxJUL) is found in the northeastern and southeastern parts, around 0.6 °C per decade (Fig. 1d). Overall trends range from 0.4 to 0.6 °C per decade for both variables.

Also in agreement with ARSO (2017), at the six chosen locations, the TmeanJJA values ranged from 18.0 °C in Postojna to 21.4 °C in Bilje and TmaxJUL from 25.5 to 29.9 °C, respectively (see Online Resource 2). The highest trend of TmeanJJA was in Novo mesto (0.5 °C/decade) and the lowest in Bilje (0.4 °C/decade), while the highest trend for TmaxJUL was in Murska Sobota (0.5 °C/decade) and the lowest again in Bilje (0.4 °C/decade). Not surprisingly, the highest number of hot days was found in Bilje, reaching on average approximately 30.8 hot days per summer. The highest trends were in Murska Sobota (3.3 hot days/decade) and in Bilje (3.2 hot days/decade). The number of summer hot days at other stations in the subcontinental climate region ranged from 15.6 (Novo mesto) to 18.5 hot days/decade (Ljubljana). In Postojna, there were on average only 7.9 hot days/decade, with the lowest trend (1.7 days/decade).

As shown in Fig. 1 and Online Resource 2, mean summer temperatures and the number of hot days increased during 1961–2011, so their respective interaction was studied. The correlation *r* values between the measured and fitted data for the quadratic fit are 0.85 (0.75–0.91) in Bilje, 0.86 (0.77–0.92) in Postojna, 0.87 (0.79–0.93) in Celje, 0.89 (0.82–0.94) in Novo mesto, and 0.91 (0.84–0.95) in Murska Sobota. In Ljubljana, the linear fit provides a correlation coefficient of 0.86 (0.76–0.92). When summer mean temperature increases 1 °C, the number of hot days increases linearly, as reflected in the slope coefficient, which ranges from 5.1 (Postojna) to around 7 (Ljubljana, Celje, Novo mesto), 8 (Murska Sobota), or even 9.5 days/°C, or even worse (following the quadratic equation).

**Fig. 2:**
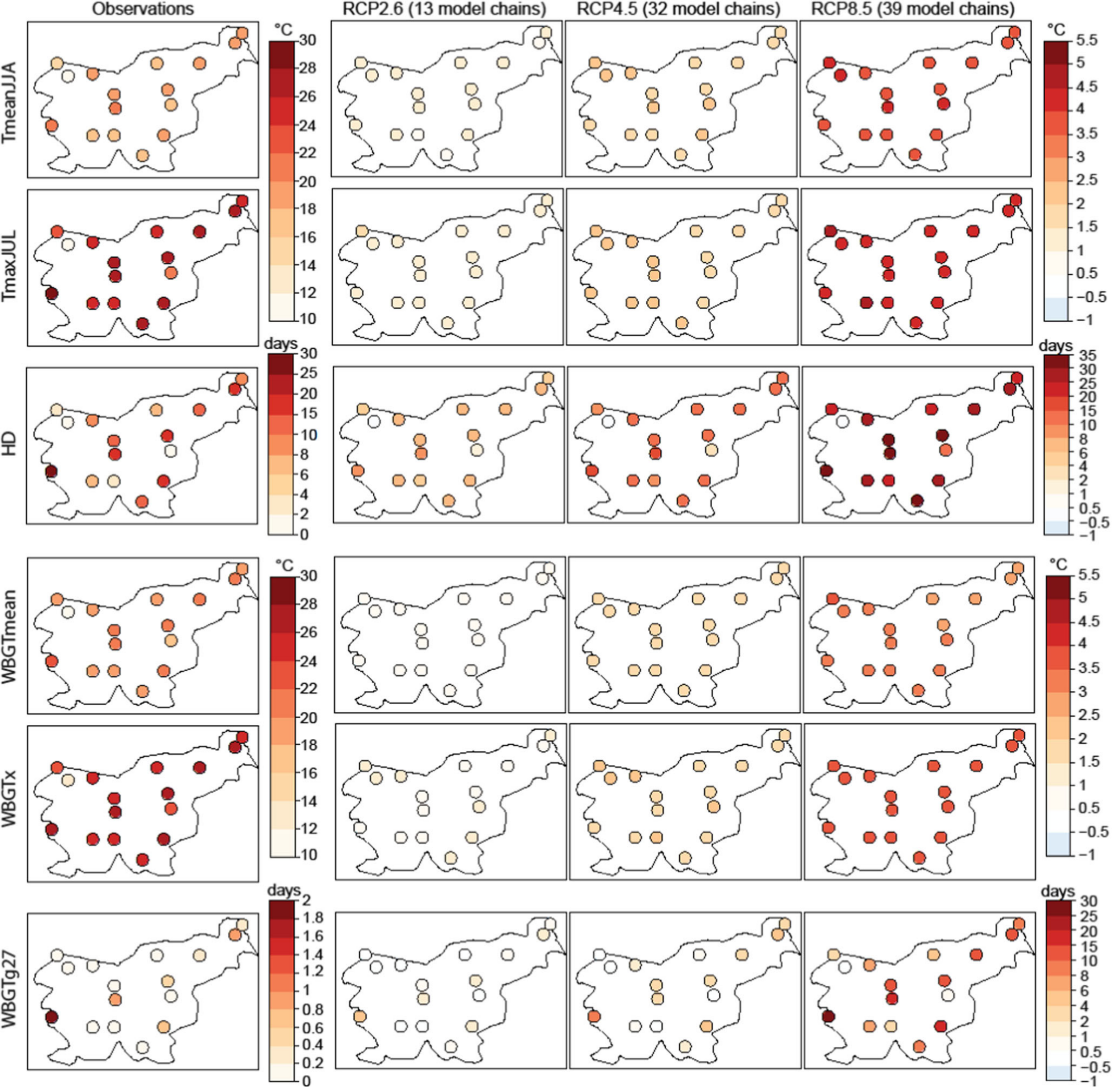
Spatial distribution of temperature- and WBGT-derived indices (see Table 1) as represented by the observations for the period 1981–2010 (first column). Climate change signal for the period 2070–2099 with respect to 1981–2010 for three RCPs (second to fourth columns). Each panel represents the multi-model ensemble median of the index and the number of RCM simulations contributing to the multi-model ensemble median is depicted in brackets

With the exception of the mountainous areas, values of heat stress (Fig. 2, first column) in all stations were between 22 and 26 °C for the summer mean (WBGTmean) and between 24 and 30 °C for the maximum index (WBGTx). Days with daily maximum heat stress above 27 °C rarely occur (on average) in present climate.

### Climate change projections of temperature and heat stress

Climate change projections of temperature and heat stress indices were produced for the Slovene locations, obtaining the climate change signal as the difference between the projections for the period 2070–2099 with respect to 1981–2010. All indices are projected to increase in Slovenia by the end of the twenty-first century and the increments vary nonlinearly with the forcing scenario (Fig. 2). For instance, changes in summer mean (TmeanJJA) and July daily maximum (TmaxJUL) range from 1 °C for the lower emission scenario (RCP2.6) to 4.5 °C for the highest emission scenario (RCP8.5) in all Slovene stations. The number of hot days (HD) might increase 2–10 days per summer under RCP2.6 and up to 35 days under the highest emission scenario. HD changes present a larger spatial variability than the other temperature indices, and they are larger in the stations with the highest TmaxJUL in present climate (Fig. 1b).

Similarly to temperature extremes, summer mean and maximum heat stress are projected to increase from 1 to 3.5 °C depending on the emission scenario in all Slovenian stations (Fig. 2, fourth and fifth rows). The frequency of extreme heat stress (WBGTg27) will be accentuated in the locations where the frequency of HD largely increase, increasing up to 20 days in the stations in the center of the country and more than 30 days in Bilje under the strongest emission scenario.

**Fig. 3:**
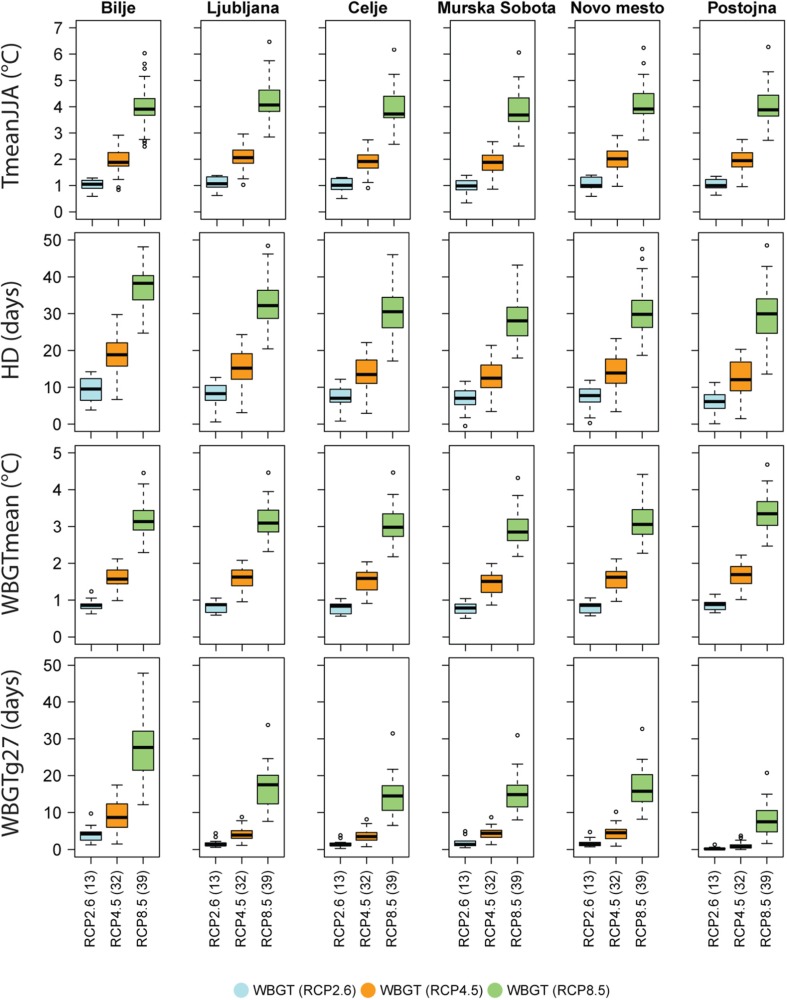
Climate change signal for some temperature- and WBGT-derived indices (rows) for six Slovene stations (columns). The boxes show the full uncertainty range across models for each RCP. The number of RCM simulations contributing to the multi-model ensemble median is depicted in brackets

Despite the model uncertainty in the climate change signal, there is overall good agreement in the mentioned changes (Fig. 3). Model uncertainty is quite similar across all stations for summer mean temperature and heat stress. In Bilje, the uncertainty in the number of days with extreme heat stress (WBGTg27) is especially large, ranging from 10 to 50 days for RCP8.5. It is interesting to see that, even under the low (RCP2.6) and moderate (RCP4.5) emission scenarios, important increases may occur in the number of hot days and high heat stress risk due to the warmer and humid conditions in the sub-Mediterranean climate region.

### Case study: the odelo factory

**Fig. 4:**
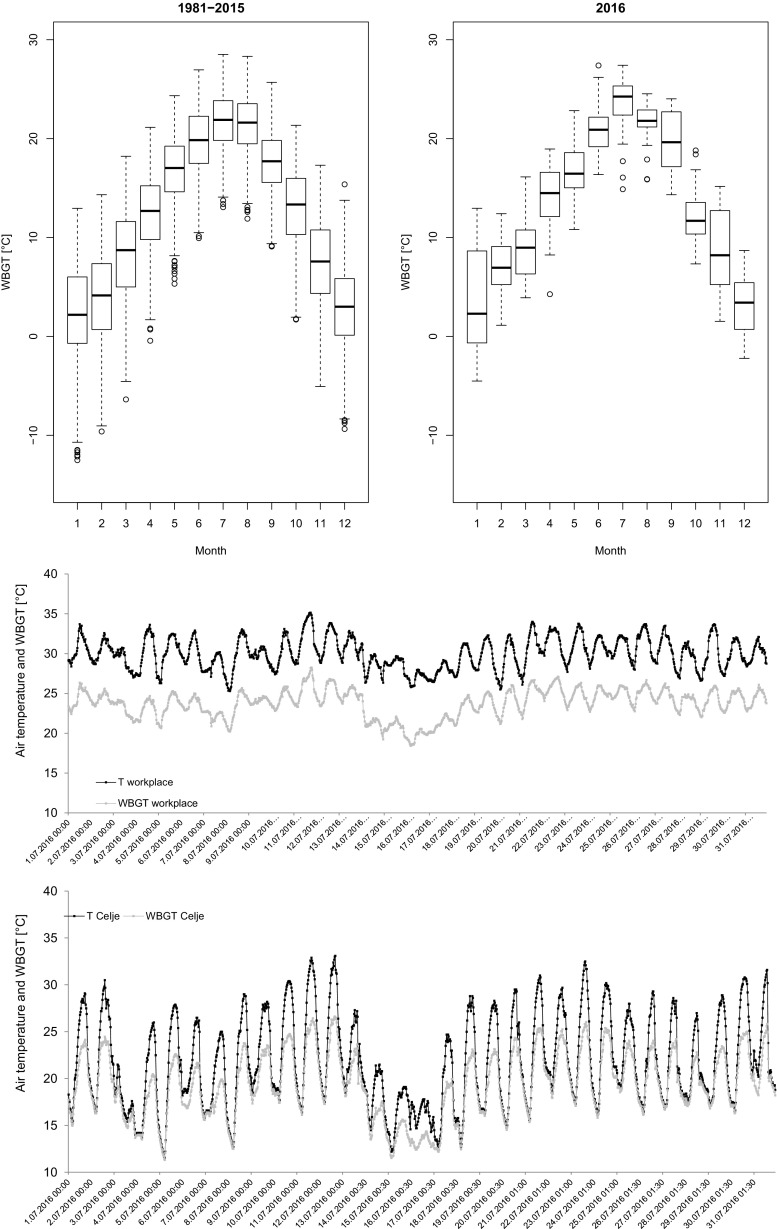
Top left panel: Monthly boxplots for daily values of in shade WBGT at Celje station for the period 1981–2015. Top right panel: Monthly boxplots for daily values of in shade WBGT at Celje station for the year 2016. Middle panel: Air temperature and indoor (1.5 m height) WBGT (every half hour) at the injection molding workplace in the odelo factory in July 2016. Bottom panel: Air temperature and in shade WBGT (every half hour) in Celje in July 2016

The first approach for an analysis of the temperature conditions in a workplace should include general information on the local temperatures during the year. For this purpose, we analyzed the data at Celje station in the period 1981–2015 and compared it with the year 2016, during which we conducted the study. Monthly values of WBGT had higher variability in winter and higher maximum values in July (Fig. 4, top left), always under 30 °C (median under 25 °C). The variability is comparable among summer months, with an interquartile range of around 4 °C. Values can, however, differ substantially from year to year. For example, in the year 2016 (Fig. 4, top right), interquartile ranges are more diverse in summer with a median of 24.2 °C in July, which is higher than the measured average in 1961–2015. Summer of 2016 was not detected as very hot, with only one heat wave in Slovenia, but still within the range of the natural variability. Thus, it can be considered representative of summer conditions in Celje.

The odelo d.o.o. company installed a ventilation system, but it cannot dissipate the excessive heat produced by the injection molding process. In July 2016, the correlation coefficient of the indoor temperatures (measured at both heights) with the temperature outside odelo was 0.85, with indoor air temperatures being higher, and with lower variability than outdoor air temperatures (Fig. 4, lower panel). As an example, even with outdoor air temperature around 15 °C, the indoor temperatures measured at 1.5 m height were between 25 and 30 °C. Relative humidity was significantly lower inside the plant compared to the outside relative humidity.

Since WBGT depends on air temperature and relative humidity, a different interaction can be observed between air temperature and WBGT at the workplace and at Celje station (Fig. 4, lower panel). At the workplace, the pattern of daily WBGT data (inside the factory) follows the pattern of the external air temperature data, with WBGT systematically around 6 °C lower. At the meteorological station (with outside air temperature and relative humidity significantly higher than inside the factory), the diurnal cycle of temperature data stretches with respect to the WBGT counterpart. Minimum daily values did not differ much, but the maximum external air temperature values were higher by about 2.4 °C. In the first half of July 2016, WBGT values at the injection molding workplace were between 20 and 25 °C. During the latter half of July, WBGT increased progressively above 25 °C, attaining maximum values of 28.3 °C.

**Fig. 5:**
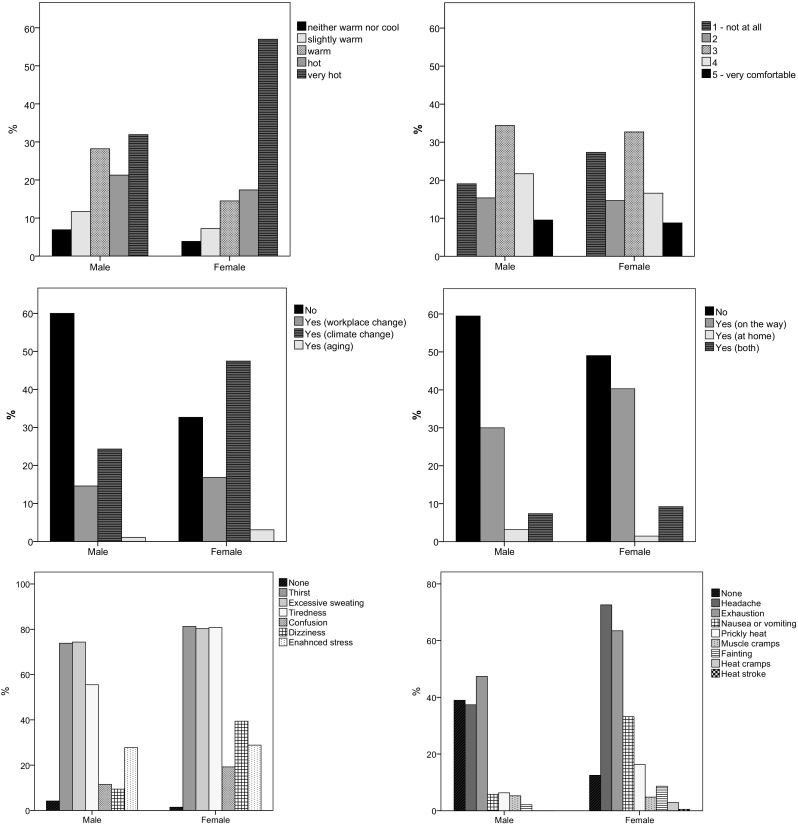
Survey conducted on workers in the odelo factory. Top left panel: How do you perceive the workplace temperature during heat waves? Top right panel: How comfortable are your clothes during heat waves? Middle left panel: Have you noticed that you have been increasingly more exposed to heat stress than in the past? Middle right panel: Is the situation regarding temperature during summer worse at home/on the way than at your workplace? Bottom left panel: Which of these heat stress symptoms do you face during work in summer? Bottom right panel: Have you ever been affected by any of these health problems at work during a heat wave?

Concomitant with the analysis of the conditions in the factory during the summer of 2016, we also surveyed the workers regarding their perception of the temperature at the workplace during heat waves. Temperature conditions were suitable for less than 4% of those completing the survey (Fig. 5, top left). For the majority, it was warm, hot, or very hot. There was a statistically significant difference (*p* < 0.001) in the number of women compared to men that perceived the working conditions as “very hot,” suggesting that they had a higher sensitivity to the hot conditions.

Working clothes were very comfortable for less than 10% of employees, with the majority of workers reporting clothing comfort between comfortable and uncomfortable (Fig. 5, top right). There was no significant difference between the males and females. For around 20% of the workers, the clothes were not comfortable at all.

The age of the workers was homogenously distributed and there was also a large group of younger workers. This needs to be considered when analyzing the questionnaires. Namely, the younger workers do not have as much experience of heat waves as the older workers. Therefore, their answers may only apply to a shorter and recent time period. Almost twice as many men than women replied that they have not been increasingly more exposed to heat stress (Fig. 5, middle left), and more than twice as many women than men replied that climate change is the main reason for experiencing heat stress (*p* < 0.001). The prevalence of women in noticing the change could be related to their higher sensitivity to heat stress conditions as shown before (Fig. 5, top left).

More than 50% of the workers reported having better climate conditions at home and on their way to work (Fig. 5, middle right), with non-statistically significant differences between men and women. However, 35% of the workers reported that their situation regarding perceived temperature was worse on the way to work than at work, with a greater percentage in women than men.

All acknowledged that heat stress can cause heat strain symptoms (Fig. 5, bottom left) leading to heat-induced illness if the problem is not resolved (Fig. 5, bottom right), which may ultimately have a fatal consequence. Since becoming operational in 2005, there has only been one incident of heat stroke in odelo and 13 incidences of heat-induced health problems that required hospitalization. Thirst and excessive sweating are the first signs of hot ambient conditions, reported by men (> 70%) and women (> 80%) in the factory (differences between males and females was not significant). Tiredness (*p* < 0.001), confusion (*p* < 0.001), and dizziness (*p* < 0.05) are more commonly perceived by women (81, 19, and 39%, respectively) than men (56, 12, and 9%, respectively). Enhanced stress due to heat is experienced by 28% of men and 29% of women.

Gender differences are also evident among the reported heat-induced health problems (Fig. 5, bottom right); 39% of the male workers did not report any health problems, whereas 37% were affected by a headache and 47% by exhaustion. These percentages were much higher for female workers, with 73% (*p* < 0.001) and 64% (*p* < 0.01), respectively. Furthermore, 33% of the women have experienced nausea or vomiting (*p* < 0.001) and 16% prickly heat (*p* < 0.01), while only 6% of the male group reported the occurrence of these symptoms. There were also cases of muscle cramps and fainting in both gender groups and in the female group also cases of heat cramps and heat stroke.

## Discussion

The Slovenian Environment Agency has reported a general trend of warming in Slovenia, particularly for summer months, during which there is an increasing trend in the number of hot days (ARSO 2017). Results of the present study are in agreement with these findings, particularly during the summer months, additionally showing positive trends of TmaxJUL and the relation between pronounced increase in the number of summer hot days (Tmax above 30 °C) and summer temperatures. Climate change projections show that mean temperature and the number of hot days will largely increase by the end of the twenty-first century, by as much as 4.5 °C and 35 days, respectively, for the high emission scenario. Mean and maximum summer values of heat stress are projected to increase over the whole country as well. Although the increase is slightly smaller than that for temperature indices, it is sufficient to increase the frequency of days with a high risk of heat stress (WBGT above 27 °C), which currently are very rare. More than 30 summer days with high heat stress conditions are projected under the higher emission scenario, especially in the sub-Mediterranean climatic area of Slovenia. It should be noted that our analyses do not consider changes in the land cover with time; thus, heat stress conditions are most likely underestimated.

During HDs, the working conditions in the manufacturing plant were near critical, with average values of the WBGT index between 20 and 25 °C, but reaching even higher values during heat waves. The survey conducted at the odelo d.o.o. factory regarding workers’ perception of thermal conditions indicated that 96% of workers perceived the temperature conditions as unsuitable.

### Heat conditions in Slovenia during summer months

Since Roman times, there has been no 30-year period on record with mean average European summer temperatures as high as observed in the last three decades (Luterbacher et al. 2016), and Slovenia is not an exception. According to Kysely (2010), the probability of occurrence of very long heat waves has risen by an order of magnitude over the recent 25 years and is likely to increase by another order of magnitude by around 2040 assuming the moderate scenario (RCP4.5) summer warming rate. As for middle and southern Europe (Thorsson et al. 2017), heat stress-related problems among the population are expected to increase as a result of climate change. Thus, the mitigation and adaptation to extreme temperature events and heat stress are of vital importance for humans during their daily activities (Basarin et al. 2016).

Climate prediction models are required to be able to translate the anticipated external conditions to the conditions within a factory. Unfortunately, this is not straightforward (Gao et al. 2017). Undoubtedly, external meteorological parameters impact on the conditions within a workplace, but it will depend on the heat being generated by the production process within the factory and the efficacy of any cooling or ventilation system installed within the factory. As a result, it is recommended to use the WBGT index or monitor air temperature and humidity at workplaces. It is well established that work capacity substantially diminishes once WBGT exceeds 26 °C (Kjellstrom et al. 2009). Thus, the WBGT index allows the determination of the work/rest cycle based on the ambient conditions within the factory. Spector and Sheffield (2014) describe the WBGT index as a relatively straightforward and acceptable heat stress index, which should be considered as the foundation for the development of any future heat stress assessments (ISO standard 7243). Unfortunately, the WBGT index does not account for the level of an individual’s activity and the clothing worn, since it is based solely on meteorological parameters (e.g., temperature, humidity, wind, and radiation). However, it can be adjusted to include factors that play an important role in heat stress (Budd 2008; ISO 2017). Fischer and Knutti (2015) found that the probability of a hot extreme at 2 °C warming is almost double than at 1.5 °C in a climate change context and more than five times higher than present-day climate. The increases of WBGT projected for Europe as a result of climate change are expected in many other regions of the world (USA, Australia, India, Caribbean) and are more predictable than increases in temperature at least in mid-latitude regions owing to the compensating effects of humidity (Sherwood and Huber 2010; Casanueva et al. 2018b).

The frequency of high heat stress values is also crucial for many working activities. To quantify this, we used the number of summer days in which WBGT is above 27 °C. Once exceeded, workers are advised to reduce working time to 15 min/h and drink at least 1 l of water per hour. On average, in current climate, this value is only reached in few of the stations included in the present analysis, with up to 2 days in Bilje. However, it is projected to increase between 10 and 30 days in most of the analyzed stations under the higher emission scenario.

Due to the potentially profound consequences of occupational heat stress on workers and on the economy in a changing climate, a re-evaluation of heat stress assessment and control strategies is necessary to prevent heat stress through the design of workplaces and communities for longer-term adaptation (Spector and Sheffield 2014). This should draw the attention of stakeholders to take action, especially in the most vulnerable areas, such as those in the (sub-)Mediterranean region.

### Case study: the odelo factory

As in many industries throughout Europe, heat stress is a common problem in the odelo factory, and workers are frequently exposed to heat stress above conventional limits (Bernard and Cross 1999). Heat stress mitigation for an acclimatized person, working at moderate work intensity (250–350 W) should start when WBGT is 26–28 °C (NIOSH 2016).

The survey conducted at odelo d.o.o. demonstrates that basic symptoms of heat stress are very common during summer and gradually progress to more serious forms, such as confusion, dizziness, enhanced stress. These can cause accidents at work, but attention should also be focused on heat-induced health problems. Our survey at the odelo factory revealed that 56% of the workers experienced headache and exhaustion, which is already a bad prognosis for future and warmer conditions. The incidences of nausea, prickly heat, muscle and heat cramps, fainting, and even heat stroke can be reduced or prevented by implementing different solutions, appropriate for the company and their working environment. In order to protect the workers from the effects of heat, some general guidelines should be followed (OSHA, Occupational Safety and Health Administration from the US Department of Labor 2017).

Most studies focusing on heat stress in industrial environments limit their observation to the work conducted in the industrial setting. In moderate climates, workers working in hot conditions can recover from the heat strain during the time away from work. Whereas under normal weather conditions heat strain may not pose a problem, during heat waves the workers may not recover properly. This may lead to workers arriving at work with some degree of heat strain still present, and this heat strain may be cumulative during the course of the heat wave. In such cases, workers might arrive at their work shift tired and unrested. They may also be experiencing heat stress at home or on the way to work. Solutions mitigating heat strain of workers must therefore also include an analysis of their conditions at home and on their way to work (Kjellstrom et al. 2011).

Since odelo company employs men and women, the effect of heat stress on both genders should also be considered. It has previously been reported that females are more sensitive to warm and cold stimuli (lower thresholds for detection of the stimulus) than men (Golja et al. 2003) and display a stronger warmth sensation to a warm stimulus (Gerrett et al. 2014). Our observations are in agreement with these results, as the female workers perceived the indoor temperatures as hotter than males. In addition to these differences in thermal sensitivity, males have been reported to have a greater sweating response (Anderson et al. 1995; Avellini et al. 1980); however, this can be attributed to morphological differences (Anderson et al. 1995; Notley et al. 2017). Matching men and women in terms of morphological characteristics results in similar cutaneous vascular and sweating responses. Cutaneous vasomotor activity of smaller individuals (mostly women but also smaller men) is greater, whereas the larger individuals depend more on sudomotor function (Notley et al. 2017). Therefore, smaller individuals might respond better to warm and humid environment, where evaporation is limited, whereas the larger individuals would respond better to hot and dry environment due to greater reliance on sweating.

Our analysis indicated that a greater portion of women perceived the heat-related tiredness, headaches, confusion, and dizziness, compared to men. This might be attributable to the traditional gender roles, which is a greater contribution of the tasks at home being conducted by females, preventing the same magnitude of recovery from heat strain as in men. The Slovene labor market is characterized by a high proportion of female employees (Kanjuo Mrčela and Černigoj Sadar 2007), and the government initiative of increasing full-time employment of females, started after the second world war. This initiative was not as successful in the private sphere (Kanjuo Mrčela and Černigoj Sadar 2007; Humer 2009; Jogan 2011). In the European Union, 25% of men and 35% of women are daily enrolled in the care and education of children, 29% of men and 79% of women do the cooking and other household tasks, while 4% of men and 9% of women engage daily in the care for the elderly (European Foundation for the Improvement of Living and Working Conditions 2009). Females therefore share an unequal burden in the domestic tasks, and this greater role in home-related tasks may prevent appropriate recovery from heat strain sustained at work. This lack of recovery will only be potentiated during heat waves and may also be the source of the gender-related difference in the responses of the odelo workers. The solution is therefore not biasing hiring practices towards one gender, but increasing the awareness of the need for equal engagement of both genders when it comes to domestic tasks.

The management in the manufacturing plant has recognized the detrimental effect of heat waves on workers’ health and well-being and also on productivity. There is an ongoing effort of increasing awareness of heat stress, and develop guidelines to minimize heat strain. Nonetheless, 22% workers stated that nothing can be done to reduce their exposure to heat stress. Drinking water to prevent dehydration and having 5-min breaks in cooler spaces every 2 h is, according to some, not sufficient to ameliorate heat strain. As elsewhere, further actions are predicated on affordability, infrastructure, and energy availability (Sherwood and Huber 2010).

## Conclusions

The results of current and emerging analyses should be the basis for effective mitigation policies in companies where heat is an escalating problem. These findings should lead to an increased awareness of heat-related risk in working environments. The present study has also demonstrated that meteorological data could be used to predict the ambient conditions in a workplace. Future developments should consider early warning systems for impending heat stress (which are also developed in the framework of HEAT-SHIELD) and appropriate solutions and strategies to mitigate the heat stress in the workplace (Kalkstein 2000).

Accepting the inevitability of climate change during this century should lead to actions safeguarding the workforce and thus also maintaining their productivity. The increased resilience of workers during heat waves will also serve to maintain the competitiveness of European industry.

## Electronic supplementary material

Online Resource 1 (PDF 184 kb)

Online Resource 2 (PDF 220 kb)
