# Supplementary material for: The effect of hot days on occupational heat stress in the manufacturing industry: implications for workers’ well-being and productivity
Source: Int J Biometeorol. 2018 Mar 30;62(7):1251–64. doi: 10.1007/s00484-018-1530-6 (PMC6028887; doi:10.1007/s00484-018-1530-6)
Supplement: Supplementary file 1 — (PDF 184 kb) [file 484_2018_1530_MOESM1_ESM.pdf]

Online Resource 1: Summary of the EURO-CORDEX simulations considered in the present work. A given combination of regional climate model driven by a global climate model for a specific realization might have been forced by one, two or three emission scenario (RCP2.6, RCP4.5 and RCP8.5) and developed at the coarse (EUR-44, at 0.44°) and/or high (EUR-11, at 0.11°) horizontal resolutions. The total number of simulations per resolution and scenario and total number of simulation per scenario are also shown.

			**RCP2.6**		**RCP4.5**		**RCP8.5**	
**Regional Climate Model**	**Global Climate Model**	**Realization**	**EUR-11**	**EUR-44**	**EUR-11**	**EUR-44**	**EUR-11**	**EUR-44**
CLMcom-CCLM4-8-17	CNRM-CERFACS-CNRM-CM5	r1i1p1			X		X	
CLMcom-CCLM4-8-17	ICHEC-EC-EARTH	r12i1p1			X		X	
CLMcom-CCLM4-8-17	MOHC-HadGEM2-ES	r1i1p1			X		X	X
CLMcom-CCLM4-8-17	MPI-M-MPI-ESM-LR	r1i1p1			X	X	X	X
CLMcom-CCLM5-0-6	CNRM-CERFACS-CNRM-CM5	r1i1p1						X
CLMcom-CCLM5-0-6	ICHEC-EC-EARTH	r12i1p1						X
CLMcom-CCLM5-0-6	MIROC-MIROC5	r1i1p1						X
CLMcom-CCLM5-0-6	MOHC-HadGEM2-ES	r1i1p1						X
CLMcom-CCLM5-0-6	MPI-M-MPI-ESM-LR	r1i1p1						X
CNRM-ALADIN53	CNRM-CERFACS-CNRM-CM5	r1i1p1			X	X	X	X
DMI-HIRHAM5	ICHEC-EC-EARTH	r3i1p1	X		X	X	X	X
HMS-ALADIN52	CNRM-CERFACS-CNRM-CM5	r1i1p1						X
KNMI-RACMO22E	ICHEC-EC-EARTH	r1i1p1			X	X	X	X
KNMI-RACMO22E	MOHC-HadGEM2-ES	r1i1p1	X	X	X	X	X	X
MPI-CSC-REMO2009	MPI-M-MPI-ESM-LR	r1i1p1	X	X	X	X	X	X
MPI-CSC-REMO2009	MPI-M-MPI-ESM-LR	r2i1p1	X	X	X	X	X	X
SMHI-RCA4	CCCma-CanESM2	r1i1p1				X		X
SMHI-RCA4	CNRM-CERFACS-CNRM-CM5	r1i1p1			X	X	X	X
SMHI-RCA4	CSIRO-QCCCE-CSIRO-Mk3-6-0	r1i1p1				X		X
SMHI-RCA4	ICHEC-EC-EARTH	r12i1p1	X	X	X	X	X	X
SMHI-RCA4	IPSL-IPSL-CM5A-MR	r1i1p1			X	X	X	X
SMHI-RCA4	MIROC-MIROC5	r1i1p1		X		X		X
SMHI-RCA4	MOHC-HadGEM2-ES	r1i1p1		X	X	X	X	X
SMHI-RCA4	MPI-M-MPI-ESM-LR	r1i1p1		X	X	X	X	X
SMHI-RCA4	NCC-NorESM1-M	r1i1p1		X		X		X
SMHI-RCA4	NOAA-GFDL-GFDL-ESM2M	r1i1p1				X		X
**TOTAL**			**5**	**8**	**15**	**17**	**15**	**24**
**TOTAL PER SCENARIO**			**13**		**32**		**39**	

Implications of climate change on the manufacturing sector in Slovenia: with particular reference to summer heat

Tjaša Pogačar[1], Ana Casanueva, Katja Kozjek, Urša Ciuha, Igor B. Mekjavić, Lučka Kajfež Bogataj, Zalika Črepinšek

International Journal of Biometeorology

[1]Tjaša Pogačar, Ph.D.
Biotechnical Faculty
University of Ljubljana
Jamnikarjeva 101, SI-1000 Ljubljana
Slovenia
E: tjasa.pogacar@bf.uni-lj.si
T: 00386 1 3203 133
