# Supplementary material for: The effect of hot days on occupational heat stress in the manufacturing industry: implications for workers’ well-being and productivity
Source: Int J Biometeorol. 2018 Mar 30;62(7):1251–64. doi: 10.1007/s00484-018-1530-6 (PMC6028887; doi:10.1007/s00484-018-1530-6)
Supplement: Supplementary file 2 — (PDF 220 kb) [file 484_2018_1530_MOESM2_ESM.pdf]

Online Resource 2: 30-year (1981–2010) averages and standard deviations of summer mean temperatures (TmeanJJA), July mean of daily maximum temperature (TmaxJUL) and summer hot days (HD), and 51-year (1961–2011) trends with 95% confidence interval for the same indices at six locations in Slovenia.

**Index**	**Location**	**Bilje**	**Ljubljana**	**Celje**	**Murska Sobota**	**Novo mesto**	**Postojna**
**TmeanJJA (°C)**	Average	21.4	20.3	19.1	19.7	19.8	18.0
	St.dev.	1.7	1.7	1.5	1.6	1.6	1.6
**TmaxJUL (°C)**	Average	29.0	27.3	26.9	27.0	26.8	25.5
	St. dev.	1.5	1.4	1.4	1.5	1.3	1.5
**HD (days)**	Average	30.8	18.5	16.9	16.6	15.6	7.9
	St. dev.	12.5	10.8	10.5	11.5	10.9	8.4
**trTmeanJJA (°C/decade)**	Average	0.4	0.5	0.5	0.4	0.5	0.5
	Conf. int.	0.3-0.5	0.3-0.6	0.3-0.6	0.3-0.6	0.4-0.7	0.3-0.6
**trTmaxJUL (°C/decade)**	Average	0.4	0.5	0.5	0.5	0.5	0.5
	Conf. int.	0.2-0.7	0.2-0.7	0.2-0.8	0.3-0.8	0.3-0.7	0.2-0.8
**trHD (days/decade)**	Average	3.2	2.9	2.5	3.3	2.7	1.7
	Conf. int.	2.8-4.1	2.6-3.7	2.4-3.4	3.1-4.2	2.7-3.6	1.7-2.7

Implications of climate change on the manufacturing sector in Slovenia: with particular reference to summer heat

Tjaša Pogačar[1], Ana Casanueva, Katja Kozjek, Urša Ciuha, Igor B. Mekjavić, Lučka Kajfež Bogataj, Zalika Črepinšek

International Journal of Biometeorology

[1]Tjaša Pogačar, Ph.D.
Biotechnical Faculty
University of Ljubljana
Jamnikarjeva 101, SI-1000 Ljubljana
Slovenia
E: tjasa.pogacar@bf.uni-lj.si
T: 00386 1 3203 133
